# Impact of Prior Mammograms on Radiologists and Radiographers' Detection of Different Breast Cancer Lesion Types

**DOI:** 10.1002/jmrs.70015

**Published:** 2025-07-28

**Authors:** Judith D. Akwo, Phuong Dung (Yun) Trieu, Melissa L. Barron, Tess Reynolds, Sarah J. Lewis

**Affiliations:** ^1^ Medical Image Optimisation and Perception Group Sydney School of Health Sciences, Faculty of Medicine and Health, The University of Sydney Camperdown Australia; ^2^ Image X Institute School of Health Sciences, Faculty of Medicine and Health, University of Sydney Camperdown Australia; ^3^ School of Health Sciences, Western Sydney University Campbelltown Australia

**Keywords:** breast cancer, mammographic features, observer performance, prior mammograms, radiographers' performance, radiologists' performance

## Abstract

**Introduction:**

Mammographic interpretation relies on the detection of suspicious abnormal changes, and reference to prior mammograms may support the detection of these changes. However, the influence of prior mammograms on the detection of different breast lesions is unclear. This paper assesses the influence of prior mammograms on the detection of different lesion types in mammograms.

**Methods:**

Mammographic test sets comprising different lesion types were interpreted in two stages. In Stage 1, eight radiologists interpreted current mammograms of 72 women (normal: *n* = 40; cancer: *n* = 32) with and without access to the prior mammograms. In Stage 2, 13 radiographers interpreted another test set containing 28 mammograms (normal: *n* = 19; cancer: *n* = 9) with and without access to the prior mammograms. Radiologists and radiographers' performances in detecting different lesion types with and without prior mammograms were compared separately using a paired t‐test.

**Results:**

In Stage 1, reference to prior mammograms did not improve sensitivity for architectural distortions (*p* = 0.48), calcifications (*p* = 0.85), discrete masses (*p* = 0.45), and non‐specific density (*p* = 0.22). However, prior mammograms improved the detection of spiculated/stellate lesions (*p* = 0.05) and diagnostic accuracy for architectural distortions (*p* = 0.006) and discrete/spiculated/stellate masses (*p* = 0.01). Prior mammograms had no impact on lesion sensitivity (*p* > 0.05). In Stage 2, no differences were observed in sensitivity and lesion sensitivity when compared to without prior mammograms for all lesion types (*p* > 0.05), but prior mammograms improved overall diagnostic accuracy (*p* ≤ 0.002).

**Conclusions:**

Prior mammograms improve the detection of spiculated/stellate lesions but have no impact on the detection of other lesion types when measuring radiologists' and radiographers' performance.

## Graphical abstract

This paper assesses the influence that prior mammograms have on the detection of different breast lesion types in mammograms. The findings show that reference to prior mammograms improved radiologists' ability to detect spiculated/stellate lesions but not other lesion types. Reference to prior mammograms did not improve radiographers' performance in detecting different types of breast lesions.

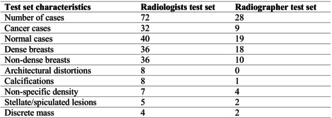

## 1 Introduction

Breast cancer remains the most prevalent cancer in women worldwide, and the incidence of the disease continues to increase annually [1]. Globally, more than three million new cases of breast cancer are predicted to be diagnosed annually by 2040 [2]. Current estimates show that one in eight women will be diagnosed with breast cancer in their lifetime, and one in 40 affected women will die from the disease [3]. In the last three decades, deaths from breast cancer have reduced by 44% [4], and this has been attributed to early detection through population‐based screening and advances in treatment of breast cancer [4]. Estimates from the United States and Australia show that screening mammography and improvement in breast cancer treatment have reduced breast cancer deaths per 100,000 women from 64 to 27 in the United States and 74 to 37 in Australia—a decrease of 37 per 100,000 women [5, 6].

Estimates show that 47% of the reduction in breast cancer deaths are due to early detection and treatment of Stages 1, 2, and 3 breast cancers [6]. Screening mammography aims to detect early‐stage breast cancers and relies on the identification of features of the disease. Across the world, radiologists and other healthcare practitioners involved in mammography interpretation use terms such as discrete mass, spiculated mass, stellate lesion, calcification, non‐specific density, and architectural distortion to describe mammographic features identified in mammograms and to report the presence of cancer [7]. These mammographic features have been shown to be correlated with histopathology features of breast cancer but may also indicate benign conditions [8, 9]. Therefore, when breast lesions are identified, they are classified according to their risk of malignancy using different lexicons such as the Breast Imaging Reporting and Data Systems (BI‐RADS) in the United States and Royal Australian and New Zealand College of Radiologists (RANZCR) classification in Australia and New Zealand [10, 11]. The initial identification and risk classification of these lesions inform screening programs' recall to assessment clinics and follow‐up.

Mammographic images are traditionally interpreted by breast radiologists. However, breast radiologists' workforce shortages have reinforced the need for breast physician and radiographer reporting. In the United Kingdom (UK) National Health Service Breast Screening Programme (NHS BSP), advanced practice radiographers undertake independent interpretation of mammograms or act as second independent double readers [12, 13]. In Australia, radiographers have demonstrated the potential to report mammograms at a level that supports their integration into the reporting workforce [14, 15, 16, 17] but have difficulty in detecting certain lesion types [14]. Previous studies based on breast radiologists have explored prior mammograms for improving the diagnostic efficacy of mammography [18, 19, 20, 21]. The rationale behind these studies is that comparing current mammograms to mammograms acquired in the previous screening rounds may facilitate the identification of parenchymal changes associated with malignancy [22, 23]. However, these studies were mostly based on older technologies and observer performance evaluation methodologies that do not consider performance at the lesion level, and only evaluated radiologists even though advanced practice radiographers, particularly in the UK NHS BSP, now participate in mammography interpretation [18].

To close these knowledge gaps, our group examined the impact that prior mammograms have on radiologists [24] and radiographers' [25] performance using current technologies and observer performance evaluation methodologies. The findings from these two studies showed that access to prior mammograms improves specificity without affecting sensitivity and lesion sensitivity, and these findings were not confounded by the experience and work‐related characteristics of radiologists and radiographers [24, 25]. However, in both studies, we observed that access to prior mammograms led to a similar trend in JAFROC, which examines performance in detecting lesions and simultaneously establishing malignancy risk [24, 25]. These findings indicated that lesion features may impact upon their risk classification and question the influence that prior mammograms have on the detection and risk classification of different lesion types. The test sets used in our previous studies included different lesion types but did not examine the impact that prior mammograms have on the detection of each lesion type [24, 25]. A recent review by our group also showed a lack of studies on the impact that prior mammograms have on radiologists' or radiographers' detection of different lesion types [18]. The only study that adjusted radiologists' performance for each lesion type was a retrospective analysis of observer performance data, and the radiologists who interpreted with prior mammograms were not always the same as those who interpreted mammograms without priors, which does not fully account for the impact that radiologists' experience has on performance [21]. Since the risk of malignancy varies across lesion types [9, 26, 27] and these risk ratings are used for reporting the presence of breast cancer in the BI‐RADS and RANZCR lexicons [10, 11], it has become increasingly important to address the knowledge gaps around the impact that prior mammograms have on performance. Therefore, this study aimed to assess the influence of prior mammograms on the detection of different breast lesion types via mammograms by radiologists and radiographers. The outcome of the work has the potential to support the design of training and continuous professional development programmes around the systematic analysis of prior mammograms for radiologists and radiographers.

## 2 Methods

This observer performance study approved by the Human Research Ethics Committee of the University of Sydney Human Research Ethics Committee (Number 2023/101) involved the interpretation of screening mammograms of women in two phases. Participants' recruitment and the interpretation protocol have been reported previously [24, 25].

### 2.1 Mammogram Test Set

Mammograms comprising of different breast lesion types/mammographic features were used to design separate test sets for radiologist and radiographers. For each participant group, two test sets were developed: ‘prior test set’ and ‘no prior test set’. The design and description of these tests and the vendor technologies used for acquiring the mammograms used for the test sets are described in our previous studies [24, 25]. The sample sizes for the test sets were based on the requirements for exploratory phase (radiographer test set) and challenge phase (radiologists test set) observer performance evaluation [28, 29]. The reference standard for the normal mammograms (mammograms of women with no cancer) was established independently by at least two expert radiologists and negative mammograms acquired 2 to 4 years later. Biopsy was used as the reference standard for the cancer cases (mammograms containing breast cancer) and expert radiologists who also established the lesion‐type descriptions. The characteristics of the test sets are summarised in Table 1. All mammograms were selected from the BreastScreen Reader Assessment STrategy (BREAST) image databank and uploaded to the BREAST platform, a cloud‐based platform technology that allows readers to interpret the mammograms and receive feedback on their performance [30, 31, 32].

**TABLE 1:** Characteristics of mammographic the test sets.

Test set characteristics	Radiologists test set	Radiographer test set
Number of cases	72	28
Cancer cases	32	9
Normal cases	40	19
Dense breasts	36	18
Non‐dense breasts	36	10
Architectural distortions	8	0
Calcifications	8	1
Non‐specific density	7	4
Stellate/spiculated lesions	5	2
Discrete mass	4	2

### 2.2 Experimental Design

The study was conducted in two stages. In the first stage, eight participants interpreted the radiologist's test set containing 72 mammograms (normal: *n* = 40; cancer: *n* = 32) in two reading sessions as described in our previous studies [24, 25]. Each reader independently interpreted the mammograms in both reading sessions based on the RANZCR breast imaging lesion classification using the BREAST platform. If a lesion was detected, the reader was asked to provide a rating from 2 to 5. A lesion rating of 2 was considered benign and non‐actionable. If a reader marked a lesion and assigned ratings of 3, 4, or 5, a list of checkboxes for different lesion‐type descriptors (spiculated/stellate lesion, discrete mass, calcification, non‐specific density, or architectural distortion) was presented for the reader to check the box corresponding to the lesion type detected. A detailed experimental design, including viewing conditions, is reported elsewhere [24, 25].

### 2.3 Statistical Analysis

Once each reader completes a test set, the BREAST software records the reader's true positives (TP), true negatives (TN), false negatives (FN), and false positives (FP) scores. The software uses these values to automatically calculate each reader's sensitivity, specificity, lesion sensitivity, receiver operating characteristic (ROC), area under the curve (AUC) and jack‐knife alternative free‐response receiver operating characteristic (JAFROC). For this study, we extracted the raw data of each reader and established their TP, TN, FN and FP for each lesion type in both reading sessions: spiculated lesion, discrete mass, calcification, non‐specific density, architectural distortion or stellate lesion. The sensitivity and diagnostic accuracy analysis for each lesion type were then performed using the MedCalc software package (Version 23.0.9) [33]. Since discrete and spiculated/stellate lesion types present as masses in mammograms, these were also combined and analysed. If a reader's mark falls within the lesion, the BREAST software records a value to indicate a correct lesion localisation. Lesion sensitivity or localisation for each lesion type was calculated by dividing the number of that lesion type correctly marked by the total number of that lesion type in the dataset. Specificity, ROC and JAFROC outputs were not analysed since the focus of the study was on the impact of prior mammograms on the detection of different lesion types and because they have been reported elsewhere [24, 25]. Kolmogorov Smirnov test showed that data for each lesion type in both the radiologists/breast physicians and radiographers' datasets were normally distributed. Therefore, the performance of the readers with and without prior mammograms for each lesion type were compared using a paired t‐test. *p* ≤ 0.05 was considered as statistically significant.

## 3 Results

The eight participants in Stage 1 routinely interpret mammograms in clinical practice. The mean number of years in their current roles was 20.3 ± 11.6 years, and the mean number of years of reading mammograms was 17.3 ± 10.9 years. These readers spent between four and 30 h interpreting mammograms and interpret 20 to 200 mammograms per week.

Table 2 shows the mean performance and the minimum and maximum scores of the participants in each lesion type when reading occurred with and without prior mammograms in Stage 1. Overall, there were no differences in sensitivity when reading occurred with compared to without prior mammograms for architectural distortions (*p* = 0.48), calcifications (*p* = 0.85), discrete masses (*p* = 0.45) and non‐specific density (*p* = 0.22). However, prior mammograms marginally improved the classification of spiculated and stellate lesions (*p* = 0.05). No differences were observed in lesion sensitivity for all lesion types (*p* ≥ 0.16). Overall diagnostic accuracy did not differ between reading with and without prior mammograms when calcifications, discrete masses, spiculated and stellate lesions and non‐specific density lesions were examined independently (*p* ≥ 0.16). However, the availability of prior mammograms significantly improved diagnostic accuracy for architectural distortions (*p* = 0.006).

**TABLE 2:** Radiologists' performance in lesion type detection with prior and no prior mammograms.

Performance metric	With priors	Without priors	*p*
Architectural distortions (*n* = 8)			
Sensitivity	58 (25–75)	62 (50–75)	0.48
Lesion sensitivity	48 (13–75)	53 (38–75)	0.49
Accuracy	86 (79–92)	74 (67–83)	0.006*
Calcifications (*n* = 8)			
Sensitivity	80 (63–100)	79 (63–100)	0.85
Lesion sensitivity	79 (63–100)	78 (63–100)	0.16
Accuracy	86 (58–100)	77 (67–85)	0.95
Discrete mass (*n* = 4)			
Sensitivity	72 (22–100)	78 (75–100)	0.45
Lesion sensitivity	68 (25–100)	69 (25–100)	0.94
Accuracy	85 (57–100)	76 (68–89)	0.18
Non‐specific density (*n* = 7)			
Sensitivity	52 (14–100)	60 (42–85)	0.22
Lesion sensitivity	48 (14–100)	57 (42–85)	0.40
Accuracy	81 (59–89)	76 (66–85)	0.29
Spiculated mass/stellate (*n* = 5)			
Sensitivity	75 (40–100)	61 (20–100)	0.05*
Lesion sensitivity	77 (40–100)	77 (20–100)	1.0
Accuracy	86 (58–100)	76 (67–87)	0.17
Discrete mass + spiculated mass/stellate masses (*n* = 9)			
Sensitivity	74 (44–100)	78 (44–100)	0.66
Lesion sensitivity	72 (44–100)	74 (22–100)	0.89
Accuracy	87 (58–100)	76 (67–87)	0.01*

*Note:* Values in brackets represent the minimum and maximum scores of the readers per lesion type.

* Significant difference.

Thirteen radiographers with mean years of experience as radiographers of 28 ± 11 years, and 21 ± 8.4 years specialised as breast radiographers completed the reading in Stage 2. All the radiographers correctly classified all the discrete masses and non‐specific density lesions when prior mammograms were available. Since only one calcification was available in the dataset, it was excluded from the final analysis. Overall, there were no differences in sensitivity and lesion sensitivity when readings occurred with compared to without prior mammograms for all lesion types (*p* > 0.05 for all). However, overall diagnostic accuracy was significantly higher for all lesion types (*p* ≤ 0.002) when prior mammograms were available to the radiographers. The mean scores of all readers, and minimum and maximum scores for each performance metric are shown in Table 3.

**TABLE 3:** Radiographers' performance in lesion type detection with prior and no prior mammograms.

Performance metric	With priors	Without priors	*p*
Discrete mass (*n* = 2)			
Sensitivity	100 (100–100)	94 (50–100)	0.35
Lesion sensitivity	100 (50–100)	88 (50–100)	0.17
Accuracy	85 (62–95)	66 (43–81)	0.002*
Non‐specific density (*n* = 4)			
Sensitivity	100 (100–100)	94 (75–100)	0.17
Lesion sensitivity	90 (75–100)	84 (50–100)	0.45
Accuracy	86 (65–96)	69 (48–83)	0.001*
Spiculated/stellate mass (*n* = 2)			
Sensitivity	75 (0–100)	75 (0–100)	1.00
Lesion sensitivity	69 (0–100)	75 (0–100)	0.59
Accuracy	83 (62–95)	64 (48–76)	< 0.001*
Discrete mass + spiculated mass/stellate masses (*n* = 4)			
Sensitivity	92 (50–100)	90 (50–100)	0.76
Lesion sensitivity	87 (50–100)	87 (25–100)	1.0
Accuracy	86 (62–95)	66 (48–83)	0.001*

*Note:* Values in brackets represent the minimum and maximum scores of the readers per lesion type.

* Significant difference.

## 4 Discussion

The findings demonstrate that prior mammograms improve radiologists' detection of spiculated/stellate lesions without impacting upon the detection of discrete masses, calcifications, non‐specific densities and architectural distortions. The results also show that prior mammograms did not have an influence on the ability of radiographers to detect different breast cancer lesion types. Diagnostic accuracy, which accounts for the reader's ability to classify both normal and abnormal mammograms, was consistently higher when prior mammograms were available to radiographers. Although the mean accuracy of radiologists for all lesion types was consistently higher when prior mammograms were available, this improvement only reached statistical significance in architectural distortions.

The correct detection and discrimination of different lesion types on mammograms are important because they influence both the false‐positive recall and false‐negative rate, which have both clinical and economic implications for screening and assessment [34]. Breast imaging readers have a mental picture of cancer features [35], and when these cancer features are detected, the lesion may likely be reported as cancer regardless of whether prior mammograms are available. This may explain the similar sensitivity and lesion sensitivity between readings with and without prior mammograms. However, different lesion types can also indicate different benign and malignant conditions [36, 37, 38], but some lesion types are more predictive of cancer than others [8, 37, 38]. For example, the risk of malignancy in discrete masses and non‐specific densities is 10% compared to 10%–50% in architectural distortions, and 75%–90% in spiculated/stellate lesions [37, 38, 39]. Our findings suggest that not all lesion types visible in mammograms are reported as malignant, even when comparison with prior mammograms suggests subtle changes in current mammograms. The discriminatory powers of lesion types may explain the lower radiologists' sensitivity and lesion sensitivity in both architectural distortions and non‐specific density lesions overall (Table 2), but higher sensitivity for spiculated/stellate lesions when prior mammograms were available. This is further supported by the results obtained when discrete masses were combined with spiculated/stellate masses, where the impact of prior mammograms on the detection of spiculated/stellate masses was attenuated.

Although the results of sensitivity analysis indicate that prior mammograms have no impact on the detection of most lesion types, the higher mean diagnostic accuracy scores with priors suggests that the availability of prior mammograms improves the discrimination between malignant and benign/normal features in mammograms. These findings highlight the influence of prior mammograms in clinical decision‐making around recall for further assessment and the management and follow‐up of patients with suspicious cancer lesions. Also, despite our use of prior mammograms obtained 2–4 years prior to allow changes in slow‐growing cancers to be visible, these changes did not improve the detection rate except for spiculated/stellate lesions. The high risk of malignancy in spiculated/stellate lesions may explain the improvement in radiologists' sensitivity in this lesion type. However, this raises concerns for radiographers who demonstrated similar sensitivity, when priors were available despite this lesion type being most predictive of breast cancer and most recalled by radiologists [39, 40]. These findings highlight the need for radiographer intervention that incorporates strategies to thoroughly analyse and compare prior and current mammograms to identify spiculated/stellate lesions and changes in this lesion type that indicate malignancy.

Previous studies that have explored the mammographic detection of different breast lesion types focussed on radiologists' sensitivity [41] or inter‐radiologist concordance in classifying breast lesion types [7, 42] and positive predictive value of different lesion types [42]. The studies that assessed the influence of prior mammograms on radiologists' performance mostly report improvement in specificity and reduction in the false‐positive rate with no impact on sensitivity and cancer detection rate [18, 43, 44]. However, none of these studies examined performance according to lesion types. The only study that attempted to assess the influence of prior mammograms on radiologists' detection of different lesion types compared prior mammograms from different vendor technologies and showed that using prior mammograms from a different vendor improved cancer detection, particularly architectural distortions compared to priors from the same vendor [21]. The authors also reported that prior mammograms from the same vendor improved the classification of normal mammograms. However, the radiologists who interpreted the mammograms with priors were not always the same radiologists who interpreted the mammograms without priors [21], and differences in the expertise of the radiologists could have influenced the results. Our test sets combined mammograms from both the same and different vendors, and the same cohort of participants read mammograms of the same women with and without priors. Therefore, our methodology considered both the impact of vendor technology and reader expertise on the influence of prior mammograms on performance in detecting different lesion types. The lack of changes in sensitivity and lesion sensitivity, but higher mean accuracy scores for readings with prior mammograms may be due to the readers performance in classifying normal mammograms when prior mammograms were available. This is supported by findings of previous studies that consistently reported higher true negatives, lower false positives and higher specificity when prior mammograms were available [18, 21, 44, 45, 46, 47, 48]. The results of our lesion‐type analyses provide further evidence that the lack of improvement in sensitivity and cancer detection rate with prior mammograms reported in previous studies [18, 21, 44, 45, 46, 47, 48] may not be due to the imaging features of breast cancer lesions.

Our study has some limitations that should be considered when interpreting the findings. First, we employed a laboratory‐based experimental study design involving cancer‐enriched mammography test sets and self‐selected radiologists and radiographers. Given that the study was a lesion‐type analysis, it was necessary for different lesion types to be adequately represented in our test sets. Second, the samples of mammograms used in the test sets, particularly the radiographer test sets, were relatively small, which may limit the generalisation of the findings. Since Australian and New Zealand radiographers do not interpret mammograms clinically, it was necessary to undertake their performance evaluation using a sample of mammograms within the range (10–50 images) scientifically recommended for exploratory baseline performance evaluation studies [28, 29]. Also, the study involved significant radiologists and radiographers' time commitment at two different time points, which underscored the need to develop test sets that have scientific merit but also mitigate the effect that fatigue may have on their performance. Thirdly, the sample of participants (radiologist/breast physicians: *n* = 8 and radiographer: *n* = 13) who completed the test sets may not represent the population of breast imaging readers. However, these numbers are within the participant sample sizes used in studies (*n* = 3 to 12 readers) that have investigated the impact of prior mammograms on reader performance, and many were highly experienced [18, 21, 44, 45, 46, 47, 48]. Although the laboratory nature of our study and the limitations highlighted may impact upon the generalisability of the findings, it should be remembered that our methodology followed the recommendations for clinical studies of observer performance in medical imaging [28, 29]. Furthermore, evidence indicates that the findings from a laboratory setting can be generalised to a clinical setting [45, 49, 50]. Therefore, the results of our study provide baseline evidence for the impact of priors in the detection of different lesion types.

## 5 Conclusions

The access to prior mammograms marginally improved the detection of spiculated/stellate lesions by radiologists but did not have an impact on the detection of other lesion types. Similarly, access to prior mammograms did not improve radiographers' ability to detect different lesion types. In this laboratory study, the higher mean diagnostic accuracy when prior mammograms were available may be due to readers' ability to correctly classify normal images rather than detect cancer, which is an important capability of screening programmes. The importance of access to prior screening images remains strong when all elements are considered, including reducing recall rates and public confidence in the balance between sensitivity and specificity of mammographic imaging.

## Ethics Statement

The study was approved by the University of Sydney Human Research Ethics Committee (Approval Number: 2023/101).

## Conflicts of Interest

The authors declare no conflicts of interest.

## Data Availability

Data used for this paper are available in the BreastScreen Reader Assessment Strategy (BREAST) database and can be made available with approval of the data custodian and the University of Sydney Human Research Ethics Committee.
